# Social Contact Patterns and Age Mixing before and during COVID-19 Pandemic, Greece, January 2020–October 2021

**DOI:** 10.3201/eid3101.240737

**Published:** 2025-01

**Authors:** Vasiliki Engeli, Sotirios Roussos, Nikolaos Demiris, Angelos Hatzakis, Vana Sypsa

**Affiliations:** National and Kapodistrian University of Athens Medical School, Athens, Greece (V. Engeli, S. Roussos, A. Hatzakis, V. Sypsa); Athens University of Economics and Business, Athens (N. Demiris)

**Keywords:** COVID-19, pandemic, coronavirus disease, SARS-CoV-2, severe acute respiratory syndrome coronavirus 2, viruses, respiratory infections, zoonoses, prevention and control, transmission, basic reproduction number, surveys and questionnaires, social distancing, Greece

## Abstract

We collected social contact data in Greece to measure contact patterns before (January 2020) and during the COVID-19 pandemic (March 2020–October 2021) and assess the effects of social distancing over time. During lockdowns, mean daily contacts decreased to 2.8–5.9 (mean prepandemic 20.4). Persons >65 years of age retained the fewest contacts during the pandemic (2.1–4.1). Compared with the first lockdown (March–April 2020), the second lockdown (November–December 2020) and third lockdown (April 2021) showed higher numbers of contacts (incidence rate ratio 1.50 [95% CI 1.27–1.76] in second lockdown and 2.19 [95% CI 1.86–2.58] in third lockdown). In 2021, an increase in contacts was apparent, which persisted during the April 2021 lockdown among persons 18–64 years of age. Our study provides evidence of the waning observance of physical distancing. Effective risk communication alongside targeted social distancing could offer alternatives to repeated lockdowns.

In the early stages of epidemics caused by emerging pathogens transmitted through respiratory or close-contact routes, social distancing has been a key strategy for mitigating transmission (*1*–*3*). Given the substantial social and economic burden of social distancing measures, quantifying their effects on transmission and how they vary by age is key. Those effects can be inferred by comparing contact patterns with and without physical restrictions. For example, the effect of school closures has been evaluated by comparing contacts from weekends and holidays to typical weekdays (*4*). The established approach for capturing mixing patterns is through empirical social contact surveys in which participants complete contact diaries with information on number of contacts and location and ages of all contacts on a given day (*5*,*6*). With the exception of a coordinated effort to assess baseline social contacts in 8 countries in Europe in 2005–2006 (*5*), most countries lack representative contact studies (*7*).

During the COVID-19 pandemic, the unprecedented, prolonged implementation of a variety of social distancing measures globally offered a unique opportunity to evaluate their effects on social contacts and to understand how the effectiveness of such restrictions might change over time in similar prolonged epidemics. Despite an increase in social contact surveys during the pandemic, geographic coverage remains limited (*8*–*10*). In addition, most of those studies were performed either in a single period or in multiple waves covering the first few months of the pandemic. Longitudinal or repeated cross-sectional surveys in representative samples over longer periods are available for only a few countries and regions, such as the United Kingdom (CoMix study until March 2022) (*11*) and the United States, Germany, and Canada (Quebec) (until 2021) (*10*,*12*,*13*). CoMix also collected data in multiple survey waves in additional countries in Europe, but most surveys have data spanning only a few months, mainly for adults, and lack baseline contact data before the pandemic (*9*). Data from repeated and longitudinal surveys suggest that the pandemic had lasting changes in social contacts in the United Kingdom, Belgium, and Netherlands, because social contacts remained lower at the end of 2022 than in prepandemic years (*14*). However, gaps remain in understanding the time-varying effects of social distancing measures throughout the pandemic, overall and by age group, and in assessing the effects of multiple lockdowns; specifically, whether those later in the pandemic had similar effects on contact patterns to those of the initial lockdown.

In Greece, repeated cross-sectional social contact surveys were conducted during 2020–2021, covering 3 lockdown periods and periods with less stringent measures. Analysis of the initial survey in early 2020 provided empirical data on social contacts in this country before the pandemic and enabled assessment of the effects of the first lockdown (*15*). This study aimed to analyze the data from all available periods to characterize and compare social contact patterns and age mixing before the pandemic, during lockdowns, and during periods with relaxed social distancing measures; to infer the effect of physical distancing measures of varying stringency on transmission; to identify determinants of the number of social contacts; and to investigate whether the effects of successive lockdowns on social contacts remained consistent throughout the pandemic.

## Methods

### Surveys

**Figure 1:**
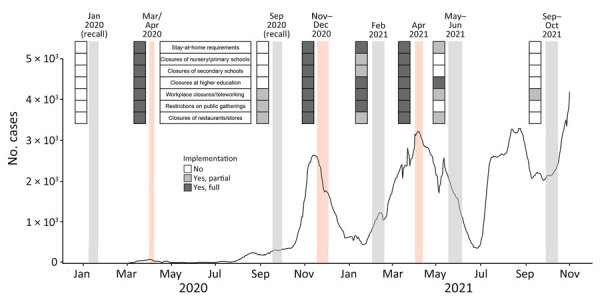
Seven-day moving average of laboratory-confirmed COVID-19 cases by date of sampling and key community measures during social contact data collection periods in study of social contact patterns and age mixing before and during COVID-19 pandemic, Greece, January 2020–October 2021. Data on COVID-19 cases were extracted from the daily reports of the National Public Health Organization. Social contact data collection periods are illustrated with shaded zones (light orange indicates lockdown periods, gray indicates prepandemic period and periods with relaxed measures). Key community measures implemented during the study periods are indicated on the left of each zone. The color of each cell represents the extent to which each community measure was implemented.

We collected information on social contacts in Greece through 6 repeated cross-sectional phone surveys with independent samples using a contact diary approach in the periods of March 31–April 7, 2020; November 17–December 3, 2020; February 1–18, 2021; April 1–12, 2021; May 17–June 5, 2021; and September 28–October 15, 2021. In the March–April 2020 and November–December 2020 surveys, participants were additionally asked to recall their contacts: participants from the March–April group were asked about contacts from mid-January 2020 (before Greece’s first confirmed COVID-19 case, thus referred to as the prepandemic period); participants from the November–December group were asked about contacts from late September 2020. In total, we collected data for 8 periods, covering 1 prepandemic period and 7 pandemic periods with varying levels of social distancing. The periods March–April 2020, November–December 2020, and April 2021 were the lockdown periods. The periods with relaxed measures were September 2020, February 2021, May–June 2021, and September–October 2021 (Figure 1). Periods were defined as lockdowns if all the following measures applied: stay-at-home requirements; closure of nursery, primary, and secondary schools and higher education; workplace closures and teleworking; restrictions in public gatherings; and closures of restaurants and stores. 

We used proportional quota sampling by age and region to recruit participants of all ages, oversampling among persons 0–17 years of age. Each survey included ≈1,200 participants throughout Greece, except for the first survey, in which we recruited 602 residents from Attica. Participants reported the number, age, and location of their contacts on the previous weekday. A contact was defined as either skin-to-skin contact or a 2-way conversation with >3 words spoken in the physical presence of another person (Appendix).

### Number of Social Contacts

We estimated the mean daily number of contacts with unique persons per participant and the corresponding 95% CI for each period. We used Cuzick’s test to assess trends over time in the number of contacts. We computed weighted estimates after adjustment for the age and sex distribution of the population of Greece by region.

### Contact Matrices and Effect of Social Distancing Measures on Transmission

We constructed age-specific contact matrices by period to capture age-mixing patterns, overall and by location, using a nonparametric bootstrap (n = 1,000 samples). We obtained the mean matrix and adjusted for the underlying demographic composition of the population and reciprocity. We estimated the anticipated relative change in the basic reproduction number, R_0_, resulting from changes in social contacts compared with prepandemic levels, using the age-specific contact matrices, as elsewhere (Appendix) (*4*,*16*).

### Effect of Lockdowns and Other Determinants on Number of Social Contacts

We fitted negative binomial generalized linear mixed (NB GLM) models with random intercepts at the individual level on the social contact data of adults and to account for repeated measurements from the same participant (in 2 surveys, participants were asked to recall contacts for additional periods). We performed variable selection (age, sex, household size, survey period, nationality, educational level, and employment status) on participants’ contact rates using Collett's algorithm (*17*) and calculated incidence rate ratios (IRRs) with corresponding 95% CIs. We included interaction terms to assess changes in the effect of explanatory variables over time and then removed if they were not significant. We present both unadjusted and adjusted results with and without the significant interaction terms.

### Sensitivity Analysis

Because the data collected in the first survey were limited to participants living in Attica, we repeated the analysis only for Attica residents. In addition, we calculated the number of contacts after censoring at 100 contacts to account for a few responses of very high daily numbers of contacts (*9*). We also fitted an NB GLM model with a more detailed age breakdown of adults and including children and adolescents, following the same approach as in the main analysis.

### Ethics Statement

Participation was voluntary, and data were collected anonymously. Participants provided oral informed consent. Children’s contacts were usually reported by a parent acting as a proxy (Appendix). The study was approved by the Ethics Committee of the Hellenic Scientific Society for the Study of AIDS, Sexually Transmitted and Emerging Diseases.

## Results

### Study Population and Number of Social Contacts

A total of 6,608 persons provided contact diaries. Of those, depending on period, 23.5%–28.1% were 0–17 years of age, 26.2%–28.9% were >65 years of age, and 51.0%–54.9% were women (Appendix Table 1).

**Figure 2:**
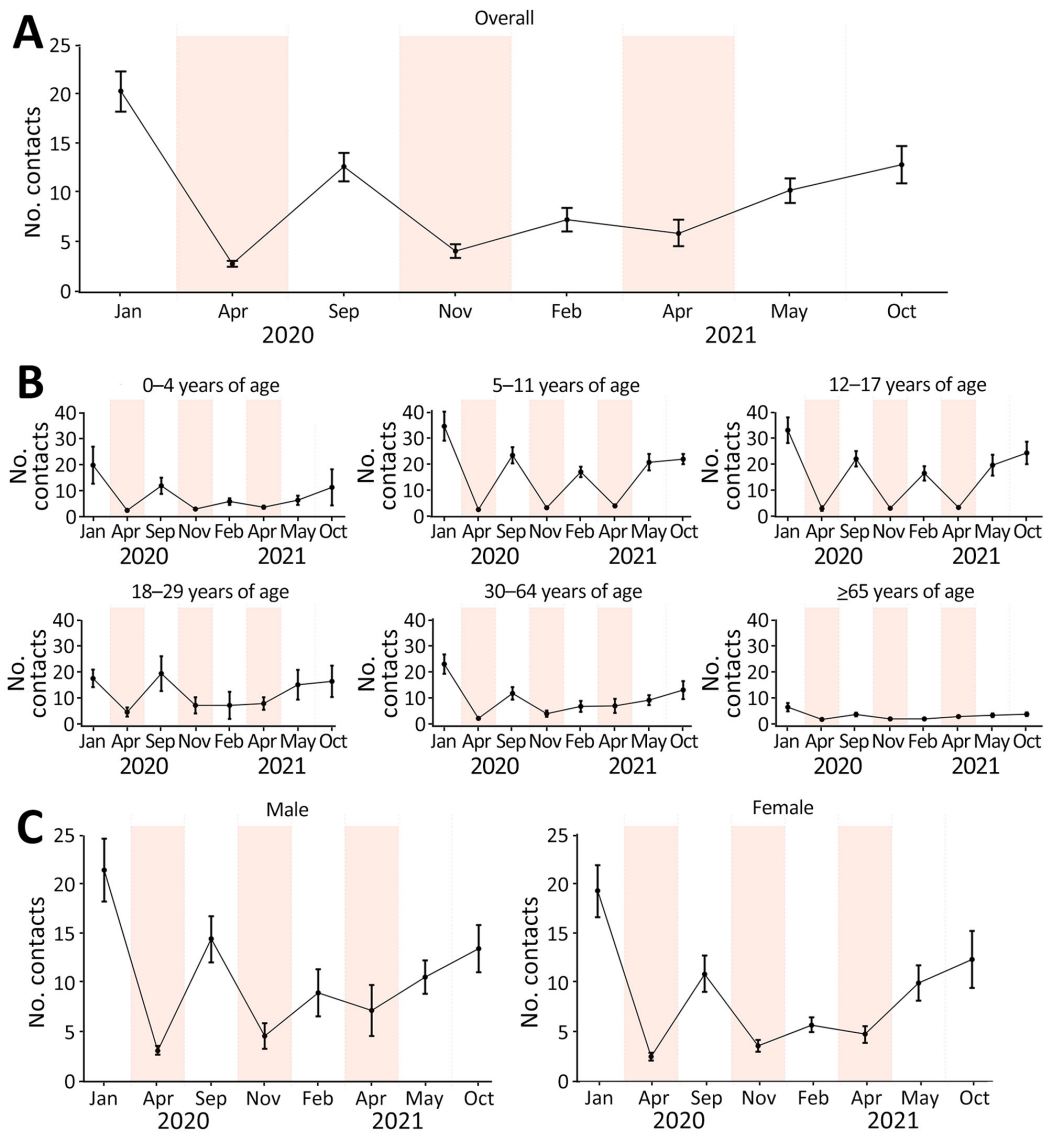
Mean daily number of recorded social contacts per participant in study of social contact patterns and age mixing before and during COVID-19 pandemic, Greece, January 2020–October 2021. Data are shown for 8 social contact data collection periods overall (A), by age group (B), and by sex (C). Estimates have been adjusted for the age and sex distribution of the population of Greece by region. Error bars mark 95% CIs. Shaded areas indicate lockdown periods.

Before the pandemic, the mean daily number of contacts per participant was 20.4 (95% CI 18.3–22.4) (Figure 2, panel A; Appendix Table 2). Throughout the pandemic survey periods, the average number of contacts remained below prepandemic levels (Figure 2, panel A). The lowest numbers of contacts were reported during lockdowns, an average of 2.8 (95% CI 2.5–3.1) in March–April 2020 (an 86.3% reduction from prepandemic), 4.1 (95% CI 3.4–4.8) in November–December 2020 (a 79.9% reduction), and 5.9 (95% CI 4.6–7.3) in April 2021 (a 71.1% reduction). The highest numbers were reported just after summer: 12.7 (95% CI 11.2–14.1) in September 2020 (a 37.8% reduction from prepandemic) and 12.9 (95% CI 11.0–14.8) in September–October 2021 (a 36.8% reduction). After censoring at 100 contacts, the mean number of contacts during the first lockdown was 2.8, during the second lockdown was 3.9, and during the third lockdown was 5.4 (Appendix Table 3).

We evaluated contact levels by location of contact across the survey periods (Appendix Table 4). We observed an increasing trend in contacts at home, work, and other settings (leisure, transport, etc.) across the 3 lockdown periods (p<0.001 for each location).

The mean number of contacts for persons 5–17 years of age was the most variable over time (Figure 2, panel B; Appendix Table 2). Children 5–11 years of age had almost identical contact levels as adolescents over time, and those levels were very high during nonlockdown periods (averaging 16.8–24.6 daily contacts). School closures during lockdowns drastically reduced daily contacts to <5. Young adults 18–29 years of age reported the highest number of contacts during lockdowns (mean 4.9–8.2), whereas elderly persons (>65 years of age) had the fewest contacts across all periods, declining from 6.8 prepandemic to 2.1–3.2 in the 3 lockdowns. After the first year of the pandemic, adult contact rates gradually increased, especially among persons 18–29 years of age. Average daily contacts for persons in that age group increased from 7.5 in February 2021 to 8.2 in April 2021, 15.4 in May–June 2021, and 16.7 in September–October 2021 (p<0.001) (Figure 2, panel B; Appendix Table 2). During the pandemic, contact rates for male participants ranged from 3.1 (95% CI 2.7–3.6) in the first lockdown to 14.5 (95% CI 12.1–16.8) in September 2020, whereas contact rates for female participants ranged from 2.5 (95% CI 2.1–2.9) in the first lockdown to 12.4 (95% CI 9.4–15.3) in September–October 2021 (Figure 2, panel C; Appendix Table 2). Similar contact patterns were estimated in the sensitivity analysis when only participants living in Attica were included (Appendix Figure 1).

### Contact Matrices

**Figure 3:**
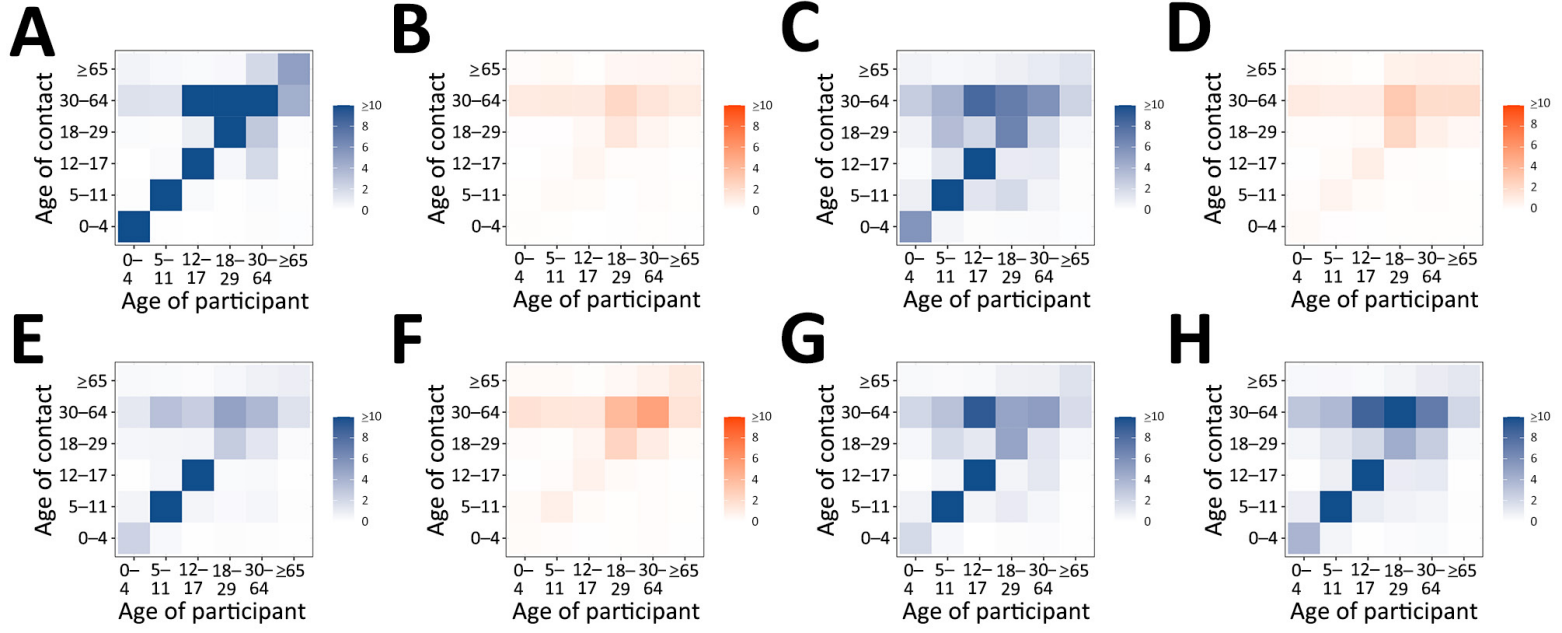
Age-specific contact matrices of all contacts in study of social contact patterns and age mixing before and during COVID-19 pandemic, Greece, January 2020–October 2021. A) January 2020; B) March–April 2020; C) September 2020; D) November–December 2020; E) February 2021; F) April 2021; G) May–June 2021; H) September–October 2021. Each cell represents the average daily number of reported contacts, stratified by the age group of the participants and their corresponding contacts. Gradient palettes were used to color contact matrices (orange indicates lockdown periods, blue indicates prepandemic period and periods with relaxed measures).

Changes in age-mixing patterns during the study period were apparent on the basis of age-stratified contact matrices (Figure 3). In the prepandemic period, we observed high levels of age assortativity (participants tended to associate more with persons of similar age), as evidenced by the diagonal of the corresponding matrix. During lockdowns, that pattern disappeared, whereas in periods with relaxed measures (including the reopening of schools), assortativity reemerged, mainly among persons of school age. The mixing of persons 30–64 years of age with persons of all ages was retained in all periods.

**Figure 4:**
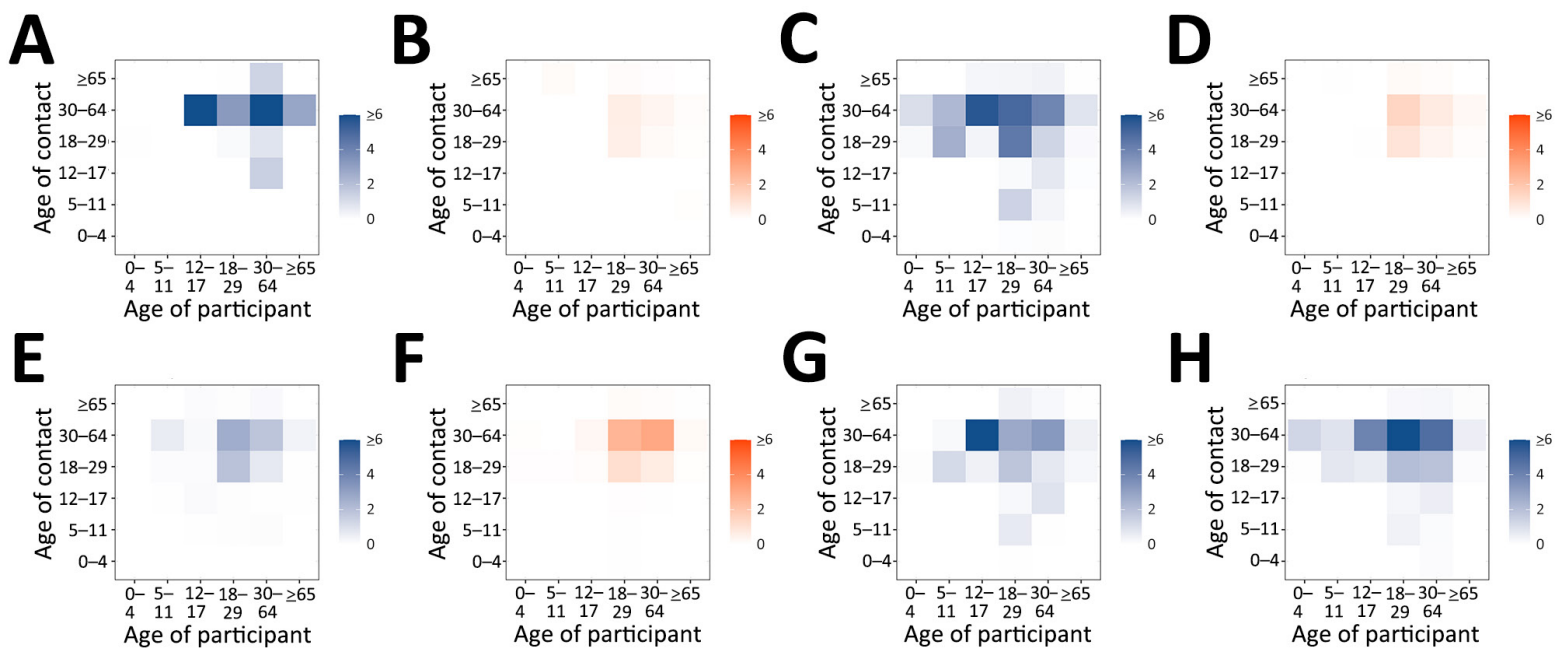
Age-specific contact matrices at work in study of social contact patterns and age mixing before and during COVID-19 pandemic, Greece, January 2020–October 2021. A) January 2020; B) March–April 2020; C) September 2020; D) November–December 2020; E) February 2021; F) April 2021; G) May–June 2021; H) September–October 2021. Each cell represents the average daily number of reported contacts, stratified by the age group of the participants and their corresponding contacts. Gradient palettes were used to color contact matrices (orange indicates lockdown periods, blue indicates prepandemic period and periods with relaxed measures).

**Figure 5:**
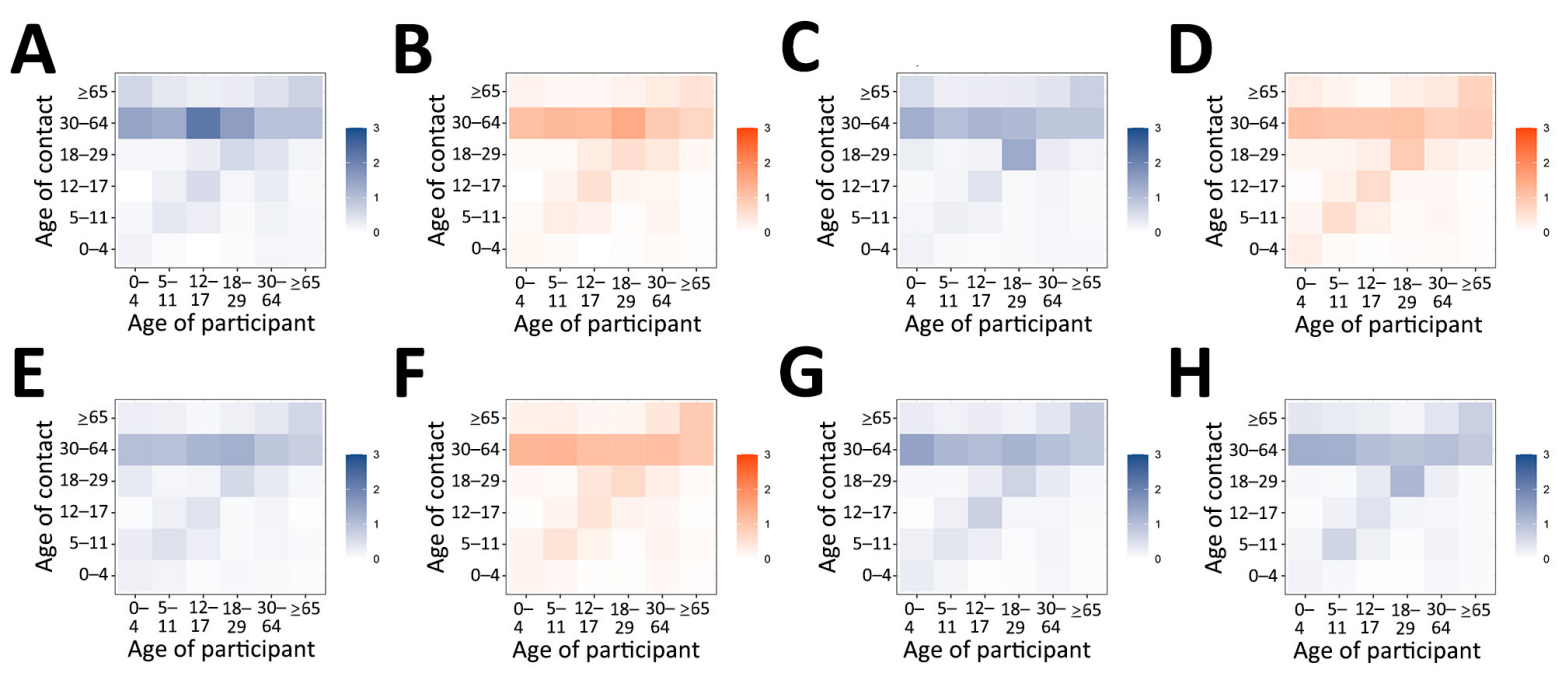
Age-specific contact matrices at home in study of social contact patterns and age mixing before and during COVID-19 pandemic, Greece, January 2020–October 2021. A) January 2020; B) March–April 2020; C) September 2020; D) November–December 2020; E) February 2021; F) April 2021; G) May–June 2021; H) September–October 2021. Each cell represents the average daily number of reported contacts, stratified by the age group of the participants and their corresponding contacts. Gradient palettes were used to color contact matrices (orange indicates lockdown periods, blue indicates prepandemic period and periods with relaxed measures).

Contact rates at work among adults decreased during lockdowns and in February 2021 more than during other periods (Figure 4), whereas age mixing at home was similar before and during the pandemic (Figure 5). Age-mixing patterns at school were comparable in the prepandemic period and during the pandemic when schools were open, whereas mixing during leisure activities did not revert to prepandemic levels (Appendix Figure 2). We also estimated the absolute difference in daily contacts between each study period during the pandemic and the prepandemic period (Appendix Figure 3).

### Effect of Social Distancing Measures on Transmission

**Figure 6:**
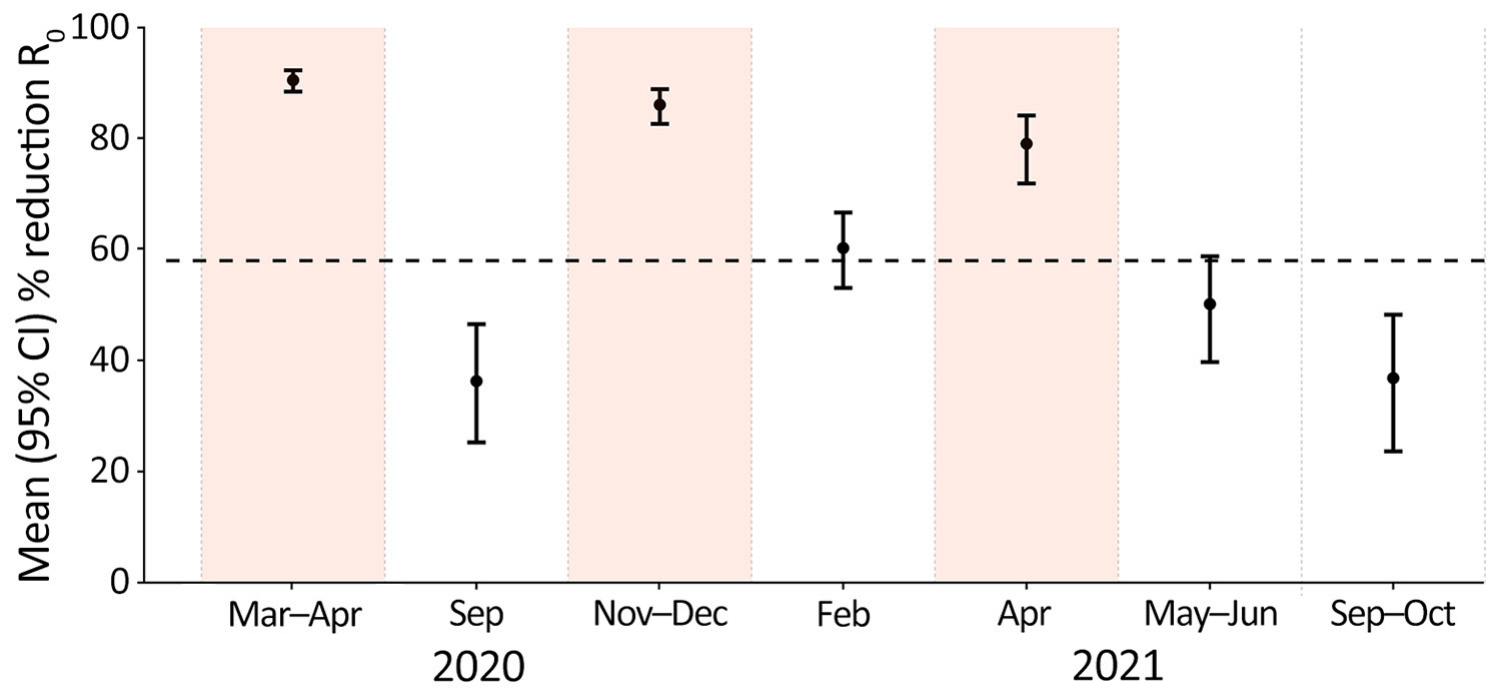
Mean reduction in R_0_ caused by physical distancing measures during COVID-19 pandemic (March 2020–October 2021) compared with prepandemic period (January 2020) in study of social contact patterns and age mixing before and during COVID-19 pandemic, Greece. R_0_ reduction was obtained by comparing social contacts data from each study period during the pandemic to the prepandemic period (January 2020). Error bars indicate 95% CIs. Shaded areas indicate lockdown periods. Dashed horizontal line indicates the minimum reduction needed to bring R_0_ to <1, assuming R_0_ is equal to 2.38 (*15*). R_0_, basic reproduction number.

Compared with prepandemic levels, the mean relative change in R_0_ resulting from changes in contact patterns was estimated to be 90.5% for the first lockdown, 86.1% for the second lockdown, and 79.1% for the third lockdown (Figure 6). Periods with relaxed measures resulted in a less pronounced reduction (36.3%–60.3%). Similar changes in R_0_ were estimated in the sensitivity analysis for Attica only (Appendix Figure 4).

### Effect of Lockdowns and Other Determinants on the Number of Social Contacts

**Figure 7:**
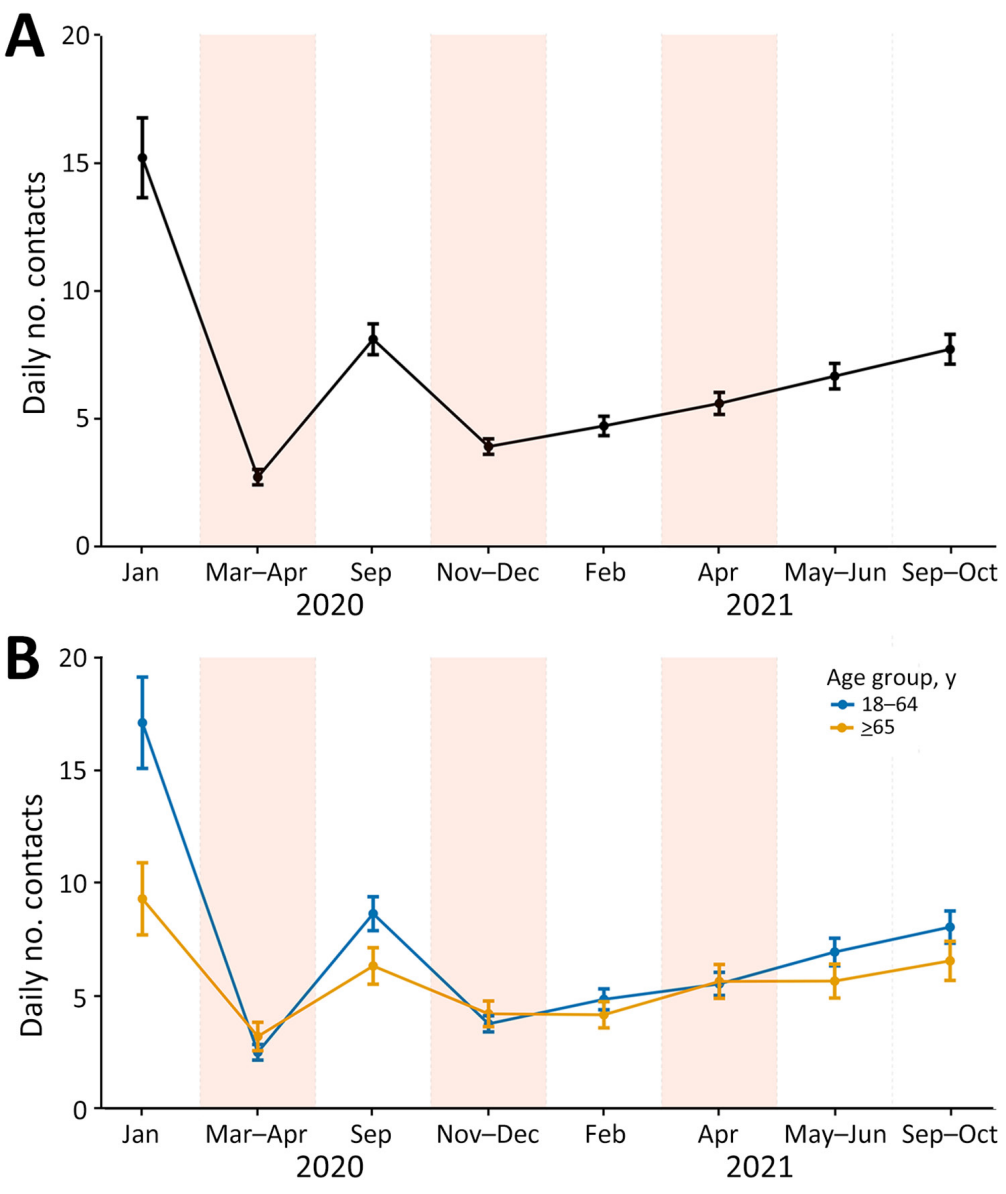
Adjusted average predictions of the number of contacts of adult participants in study of social contact patterns and age mixing before and during COVID-19 pandemic, Greece, January 2020–October 2021 (N = 6,270). Data are shown for A) study period and B) study period according to the age group of participants. Results from negative binomial generalized linear mixed models with random intercepts at the individual level fitted on social contact data collected across 8 periods in Greece through cross-sectional surveys. Error bars indicate 95% CIs. Shaded areas indicate lockdown periods.

On the basis of our analysis using the NB GLM model, time period affected contact rates among adults (Table; Figure 7, panel A). The number of contacts increased with each subsequent lockdown (second lockdown IRR = 1.50 [95% CI 1.27–1.76]; third lockdown IRR = 2.19 [95% CI 1.86–2.58]) (Table; Figure 7, panel A). The same trend was observed when the analysis was repeated exclusively among adults living in Attica (Appendix Figure 5). After the first year of the pandemic, an upward trend was apparent among adults, even though a lockdown was implemented in April 2021. We observed an interaction effect between age group and study period; for nonlockdown periods, we observed a higher number of contacts for persons 18–64 years of age than for elderly persons, whereas during lockdown periods, similar contact rates were observed for those 2 age groups (Table; Figure 7, panel Β).

**Table:** Predictors of the number of social contacts of 6,270 adult participants in study of social contact patterns and age mixing before and during COVID-19 pandemic, Greece, January 2020–October 2021*

Covariate	Unadjusted			Adjusted				
				Without interaction term			With interaction term	
	IRR (95% CI)	p value		IRR (95% CI)	p value		IRR (95% CI)	p value
Age group, y		<0.001			<0.001			0.046
18–64	Referent			Referent			Referent	
>65	0.47 (0.44–0.51)			0.86 (0.80–0.93)			1.28 (1.00–1.62)	
Sex		<0.001			0.021			0.018
M	Referent			Referent			Referent	
F	0.80 (0.75–0.86)			0.93 (0.88–0.99)			0.93 (0.88–0.99)	
Household size, including participant								
1	Referent			Referent			Referent	
2	1.35 (1.23–1.49)	<0.001		1.34 (1.23–1.46)	<0.001		1.34 (1.23–1.46)	<0.001
3	1.83 (1.64–2.04)	<0.001		1.56 (1.41–1.72)	<0.001		1.56 (1.41–1.72)	<0.001
4	2.55 (2.25–2.88)	<0.001		2.00 (1.79–2.23)	<0.001		2.00 (1.79–2.23)	<0.001
>5	3.19 (2.62–3.88)	<0.001		2.63 (2.22–3.12)	<0.001		2.63 (2.22–3.12)	<0.001
Nationality		0.010			<0.001			<0.001
Greek	Referent			Referent			Referent	
Other	0.73 (0.58–0.93)			0.65 (0.53–0.80)			0.65 (0.53–0.79)	
Time period								
January 2020, prepandemic	5.25 (4.70–5.87)	<0.001		5.22 (4.67–5.82)	<0.001		6.75 (5.92–7.69)	<0.001
March–April 2020†	Referent			Referent			Referent	
September 2020	2.46 (2.14–2.84)	<0.001		2.88 (2.52–3.28)	<0.001		3.42 (2.91–4.01)	<0.001
November–December 2020†	1.23 (1.07–1.43)	0.004		1.45 (1.27–1.66)	<0.001		1.50 (1.27–1.76)	<0.001
February 2021	1.39 (1.20–1.61)	<0.001		1.71 (1.49–1.95)	<0.001		1.92 (1.63–2.27)	<0.001
April 2021†	1.70 (1.47–1.96)	<0.001		2.07 (1.81–2.36)	<0.001		2.19 (1.86–2.58)	<0.001
May–June 2021	2.03 (1.76–2.35)	<0.001		2.40 (2.10–2.74)	<0.001		2.75 (2.34–3.23)	<0.001
September–October 2021	2.28 (1.98–2.63)	<0.001		2.78 (2.43–3.17)	<0.001		3.18 (2.71–3.74)	<0.001
Educational level								
Up to junior high school	Referent			Referent			Referent	
Up to general/vocational lyceum	1.61 (1.47–1.77)	<0.001		1.21 (1.11–1.32)	<0.001		1.22 (1.12–1.33)	<0.001
Higher education	2.04 (1.85–2.24)	<0.001		1.34 (1.23–1.46)	<0.001		1.34 (1.23–1.46)	<0.001
Employment status		<0.001			<0.001			<0.001
Not employed	Referent			Referent			Referent	
Employed	2.66 (2.49–2.83)			2.00 (1.86–2.16)			1.99 (1.85–2.14)	
Age group >65 × survey period								
January 2020, prepandemic							0.43 (0.34–0.54)	<0.001
September 2020							0.57 (0.44–0.76)	<0.001
November–December 2020†							0.88 (0.66–1.16)	0.353
February 2021							0.67 (0.51–0.89)	0.006
April 2021†							0.80 (0.60–1.06)	0.115
May–June 2021							0.64 (0.48–0.84)	0.002
September–October 2021							0.64 (0.48–0.84)	0.002

*Results from negative binomial generalized linear mixed models with random intercepts at the individual level fitted on social contact data collected across 8 periods in Greece through cross-sectional surveys. IRR, incidence rate ratio. †Lockdown period.

We identified additional independent predictors of the number of social contacts among adults (Table). Women had a lower number of contacts than did men (IRR = 0.93 [95% CI 0.88–0.99]), as did participants who were not of Greek nationality (other nationality vs. Greek nationality IRR = 0.65 [95% CI 0.53–0.79]). The number of contacts increased with larger household size or higher educational level. Compared with unemployed persons, employed persons reported a higher number of contacts (employed vs. unemployed IRR = 1.99 [95% CI 1.85–2.14]).

In the sensitivity analysis, which included children and adolescents, we noted an interaction effect between age group and study period. During nonlockdown periods, the highest number of contacts was observed among children and adolescents, followed by adults <64 years of age; elderly persons had the lowest number of contacts. During lockdown periods, contact rates were relatively similar across all age groups, with the exception of the third lockdown, in which persons 18–64 years of age reported higher contacts than children, adolescents, and elderly persons (Appendix Figure 6). After the third lockdown in April 2021, the largest increase in the number of contacts was observed among children and adolescents 0–17 years of age.

## Discussion

This study reports findings from repeated social contact surveys conducted in Greece, covering 1 prepandemic period and 7 periods during the pandemic. Before the pandemic, contact rates were notably high, comparable to those reported in another country in southern Europe (*5*). During the pandemic, daily contact rates decreased substantially (71.1%–86.3% during lockdowns and 36.8%–64.2% during periods with relaxed measures), and we observed changes in age-mixing patterns. Similar marked reductions in social contacts during lockdowns, particularly during March–April 2020, have been reported elsewhere (*6*,*8*,*10*,*16*,*18*). Young adults 18–29 years of age reported the highest number of contacts during lockdowns, whereas elderly persons maintained the lowest contact rates throughout the pandemic (lower than prepandemic levels), as reported in other studies (*6*,*8*,*19*). Overall, contacts remained below prepandemic levels throughout the study period, in accordance with other studies with data through 2021 or 2022 (*6*,*10*,*12*,*14*). Contacts increased with each subsequent lockdown and across all settings (home, work, other). The number of contacts also gradually increased after the first year of the pandemic, in particular among adults 18–64 years of age, persisting even during the third lockdown in April 2021. The CoMix survey in the United Kingdom also included data over a period covering 3 lockdowns (*6*). In contrast to our findings, contact rates among adults 18–59 years of age in the United Kingdom during the third lockdown (January–March 2021) were similar to or lower than those during the first lockdown in spring 2020.

The finding of waning observance of physical distancing policies among adults after months of mitigation measures in Greece could be attributed to multiple factors. Early in the pandemic, the World Health Organization highlighted the issue of pandemic fatigue (*20*). The observed increasing trends might also reflect previous infection, practical needs (e.g., in-person work), mask use, and vaccination uptake. Because mask mandates in Greece were already in place at the time of the September 2020 survey, they are unlikely to have contributed to the observed increasing trends. Of note, the identified increase in contact levels with each subsequent lockdown does not seem to result from increased vaccine uptake, because vaccines were not available in the second lockdown and coverage was very low among those <60 years of age in the third lockdown (Appendix Table 5). Vaccine coverage among children remained low throughout the study periods, and substantial coverage among young adults was only evident in the final survey.

Men reported higher numbers of contacts than women did during the pandemic, as seen in other studies (*21*,*22*). A larger household, higher educational level, being employed, and Greek nationality were also associated with higher contact rates. The association of higher educational level with higher contact rates aligns with existing literature suggesting that persons with higher socioeconomic status, as measured by education and employment, tend to have more social contacts (*23*). The observed variations surrounding nationality could be attributed to various factors, such as limited social networks for persons not of Greek nationality (because of homophily), underreporting because of fear of disclosing contacts when restrictions were applied, and type of employment. A similar pattern was identified in Luxembourg, where persons of most foreign nationalities reported fewer contacts (*24*).

Physical distancing measures, particularly school closures, significantly reduced age-assortative social mixing, in line with findings from other surveys (*8*). Persons 30–64 years of age interacted with persons of all ages regardless of social distancing. Given their role as bridge between children and elderly persons, encouraging masking and vaccination in this age group is key for protecting vulnerable populations from respiratory illnesses.

Physical distancing measures imposed during lockdowns are likely to have a substantial effect on transmission, with a reduction of R_0_ of 79.1%–90.5%. Less stringent physical restrictions are expected to result in a more moderate decline of 36.3%–60.3%. Those findings suggest that lockdowns can effectively suppress the R_0_ below 1.0 in epidemics with R_0_ values as high as 4.8, potentially even as high as 10.5. With less stringent measures, a decrease below 1.0 might be achievable for outbreaks with R_0_ up to 1.5 or 2.5.

A strength of this study is the longitudinal assessment of social contacts in representative samples over an extended period during the pandemic, which included multiple lockdowns. Our study builds on earlier research by examining changes in adherence to physical distancing policies over time and exploring age-specific trends in a country in southern Europe with high prepandemic contact rates. Studies on this topic are needed because variations exist among countries in baseline rates of social contact and in factors influencing adherence to physical distancing, such as political trust (*25*). In this empirical social contact study on mixing patterns in Greece before and during the pandemic, the same design, questionnaire, sampling and recruitment methodology, and market research company were used throughout the survey periods. Another contact survey conducted in Greece mainly among adults covered a relatively short period during the pandemic (February–June 2021) (*9*). In contrast to other studies that rely on historical contact data or, in the absence of empirical contact surveys, on synthetic contact data (*6*,*14*,*26*), our analysis used prepandemic contact patterns assessed by asking respondents to recall their contacts just before the pandemic, as done elsewhere (*27*). Moreover, we oversampled children and adolescents to derive more accurate insights into the contact patterns of the young population. Those data can inform policy decisions regarding those age groups (e.g., school closures).

The first limitation of our study is that self-reported social contacts are susceptible to bias (overreporting or underreporting) because of inaccurate recall or social desirability effects, particularly given that some social distancing measures were mandated during the study periods. Bias because of inaccurate recall is more relevant for January and September 2020, for which data were collected retrospectively. Furthermore, the previous weekday might not have been a typical day for all respondents. Another limitation is that contact data collected by paper diaries tend to be more complete than computer-assisted telephone interviews (*28*). Because telephone interviews were used across all our surveys, this factor should not have affected identified time trends. Telephone surveys enable a better representation of the population than online diaries or apps, which often undersample children and elderly persons. Because the definition of a contact was described simply to participants, age or educational level are unlikely to have affected the understanding of the question. Children’s contacts were usually collected through a parent acting as a proxy, which could have led to inaccurate reporting. Not all persons invited to participate in the survey did so, suggesting a potential for selection bias. Finally, although we intended to describe contact patterns representative of the entire country, the initial survey, which was conducted during the first lockdown, was limited to a smaller sample from Attica because of the urgency of the novel pandemic and the uncertainty surrounding the duration of lockdown. The results from the sensitivity analysis indicate that contact patterns in Attica were consistent with those obtained using the total sample (Appendix).

We assume that direct contacts are a proxy for social contacts that are effective for transmission. However, the mandatory mask use policy potentially decreased the number of effective contacts (*29*). In addition, widespread implementation of self-testing in workplaces and schools was introduced in mid-to-late April 2021 in Greece (i.e., in the period covered by the 2 last surveys). Therefore, the observed increase in contacts during phases of the study period might not necessarily translate to a corresponding increase in transmission.

In conclusion, our study confirms the marked decrease in social contacts during lockdown periods and provides evidence of the waning observance of physical distancing policies after several months of mitigation measures in Greece, particularly among persons 18–64 years of age and among children and adolescents when schools were open for in-person learning. However, the substantial effect on R_0_ estimated even during periods with eased restrictions and the consistently low contact rates among elderly persons, even 19 months after the onset of the pandemic, suggest that alleviating the burden of emerging epidemics without resorting to prolonged lockdowns, which incur substantial economic and social repercussions and disrupt the education process, might be feasible.

Appendix Additional information about social contact patterns and age mixing before and during COVID-19 pandemic, Greece, January 2020–October 2021.
