# Supplementary material for: Social Contact Patterns and Age Mixing before and during COVID-19 Pandemic, Greece, January 2020–October 2021
Source: Emerg Infect Dis. 2025 Jan;31(1):75–85. doi: 10.3201/eid3101.240737 (PMC11682816; doi:10.3201/eid3101.240737)
Supplement: Appendix — Additional information about social contact patterns and age mixing before and during COVID-19 pandemic, Greece, January 2020–October 2021. [file 24-0737-Techapp-s1.pdf]

Article DOI: https://doi.org/10.3201/eid3101.240737

EID cannot ensure accessibility for supplementary materials supplied by authors. Readers who have difficulty accessing supplementary content should contact the authors for assistance.

Social Contact Patterns and Age-Mixing before and during COVID-19 Pandemic, Greece, January 2020–October 2021

Appendix

Social Contacts Surveys

We conducted 6 social contact surveys during the COVID-19 pandemic in Greece covering 8 periods. Proportional quota sampling was used to recruit participants of all ages with oversampling among persons 0–17 years of age. Quotas were based on the age of the participants and the first-level NUTS (NUTS 1) regions of Greece. NUTS (Nomenclature of territorial units for statistics) has been developed in European Union to reference countries' regions for statistical purposes and NUTS 1 divides each European Union country into major socio-economic regions. In Greece, there are 4 NUTS 1 regions.

The data were collected through phone interviews conducted by trained staff using the method of computer-assisted telephone interviews (CATI). Random digital dialing was used to reach the population. Only one person in each household was asked to participate in each study. Calls were placed between 10:00 a.m.–3:00 p.m. and 5:30 p.m.–9:30 p.m. to ensure that employed persons, persons of school age etc. could be reached.

The questionnaire consisted of two sections: 1) general information, such as age, sex, place of residence, number of household members, educational level, employment status, and nationality, and 2) a contact diary for a 24-hour period from 5:00 a.m. of the day before the interview to 5:00 a.m. of the day of the interview (or Friday if interviewed on Monday). In the first and second surveys, in which participants were additionally asked to recall their contacts

from mid-January 2020 (pre-pandemic) and late September 2020 (after the opening of schools and before the Greek government determined levels of preventive measures and rules for each regional unit in Greece), respectively, there was a third section that included a contact diary for the same day of the week in those periods. Participants were asked to list each contact (up to 40 contacts) and their characteristics separately ("individual contacts"). Participants also had the option to report aggregated numbers of additional contacts they were not able to list individually ("group contacts"). Participants were asked to report each contact person only once per day (contacts with unique people). The information collected included the age and location (home, school, work, transport, leisure, or other) of the contact.

Effect of Social Distancing Measures on Transmission

The anticipated relative change in the basic reproduction number, R_0, resulting from changes in social contacts compared to prepandemic levels, was obtained by calculating the ratio of the dominant eigenvalues of the corresponding social contact matrices. We obtained the corresponding 95% CIs using nonparametric bootstrap (n = 1,000) on the data.

Of note, those estimates on the effect of social distancing measures on R_0 are theoretical and do not account for the different susceptibility and infectivity of the various variants.

Consent and Data Collection for Children and Adolescents

Parental-proxy completion was used for all children 0–11 years of age and for children and adolescents 12–17 years of age if the parent did not consent to provide information on their own. More specifically, interviews of persons <18 years of age were performed as follows: parents or guardians responded to the questionnaire on behalf of children 0–11 years of age; for children and adolescents 12–17 years of age, either the participant provided information on their own with parental informed consent, or parents provided information on behalf of the participant. For parental-proxy completion, parents were asked to collaborate with their child if the child was old enough to provide information.

Sociodemographic Characteristics of the Participants

Appendix Table 1. Sociodemographic characteristics of the participants in the 6 social contact surveys during the COVID-19 pandemic, Greece, March 2020–October 2021

	No. (%) participants					
Characteristic	March–April 2020, n = 602	November–December 2020, n = 1,203	February 2021, n = 1,200	April 2021, n = 1,201	May–June 2021, n = 1,202	September–October 2021, n = 1,200
Age group, y						
0–4	20 (3.3)	51 (4.2)	61 (5.1)	61 (5.1)	56 (4.7)	52 (4.3)
5–11	58 (9.6)	102 (8.5)	141 (11.8)	125 (10.4)	104 (8.7)	132 (11.0)
12–17	83 (13.8)	130 (10.8)	135 (11.2)	133 (11.1)	130 (10.8)	125 (10.4)
18–29	74 (12.3)	159 (13.2)	115 (9.6)	150 (12.5)	168 (14.0)	165 (13.8)
30–64	209 (34.7)	413 (34.3)	415 (34.6)	402 (33.5)	418 (34.8)	401 (33.4)
≥65	158 (26.2)	348 (28.9)	333 (27.8)	330 (27.5)	326 (27.1)	325 (27.1)
Sex						
M	295 (49.0)	567 (47.1)	580 (48.3)	548 (45.6)	542 (45.1)	582 (48.5)
F	307 (51.0)	636 (52.9)	620 (51.7)	653 (54.4)	660 (54.9)	618 (51.5)
Household size (including participant)						
1	81 (13.5)	144 (12.0)	190 (15.8)	155 (12.9)	155 (12.9)	194 (16.2)
2	185 (30.7)	454 (37.7)	390 (32.5)	426 (35.5)	417 (34.7)	421 (35.1)
3	147 (24.4)	263 (21.9)	246 (20.5)	281 (23.4)	286 (23.8)	256 (21.3)
4	148 (24.6)	242 (20.1)	266 (22.2)	241 (20.1)	248 (20.6)	237 (19.8)
≥5	41 (6.8)	100 (8.3)	108 (9.0)	98 (8.2)	96 (8.0)	92 (7.7)
Place of residence						
Attica	602 (100.0)	426 (35.4)	436 (36.3)	419 (34.9)	436 (36.3)	432 (36.0)
Thessaloniki	0 (0.0)	125 (10.4)	88 (7.3)	87 (7.2)	96 (8.0)	86 (7.2)
Other regions	0 (0.0)	652 (54.2)	676 (56.3)	695 (57.9)	670 (55.7)	682 (56.8)
Educational level*						
Up to junior high school	46 (10.4)	197 (21.4)	203 (23.5)	210 (23.8)	164 (18.0)	185 (20.8)
Up to general/ vocational lyceum	180 (40.8)	397 (43.2)	315 (36.5)	311 (35.3)	371 (40.7)	339 (38.0)
Higher education	213 (48.3)	326 (35.4)	341 (39.5)	357 (40.5)	371 (40.7)	365 (41.0)
DA	2 (0.5)	0 (0.0)	4 (0.5)	4 (0.5)	6 (0.7)	2 (0.2)
Employment status*						
Not employed	192 (43.5)	547 (59.5)	535 (62.0)	538 (61.0)	548 (60.1)	529 (59.4)
Employed	248 (56.2)	373 (40.5)	326 (37.8)	333 (37.8)	360 (39.5)	356 (40.0)
DA	1 (0.2)	0 (0.0)	2 (0.2)	11 (1.2)	4 (0.4)	6 (0.7)

*Adult participants only.

Number of Contacts by Survey Period

We computed weighted estimates for the number of social contacts after adjustment for the age and sex distribution of the population of Greece based on the first-level NUTS regions, to limit the potential lack of representativeness of the study population. We grouped the participants' place of residence into 3 categories: Attica (which includes Athens; the largest city and capital of Greece), Thessaloniki (the second largest city of Greece) and other regions.

Appendix Table 2. Mean (95% CI) daily number of contacts per participant by survey period, Greece, January 2020-October 2021*

Study Period	January 2020	March–April 2020	September 2020	November–December 2020	February 2021	April 2021	May–June 2021	September–October 2021
No. participants	602	602	1,203	1,203	1,200	1,201	1,202	1,200
Overall	20.4 (18.3–22.4)	2.8 (2.5–3.1)	12.7 (11.2–14.1)	4.1 (3.4–4.8)	7.3 (6.1–8.5)	5.9 (4.6–7.3)	10.3 (9.0–11.5)	12.9 (11.0–14.8)
Age group, y								
0–4	19.9 (12.7–27.0)	2.6 (2.1–3.0)	12.0 (8.9–15.1)	3.1 (2.8–3.4)	6.0 (4.7–7.2)	3.8 (3.2–4.4)	6.5 (4.7–8.2)	11.4 (4.5–18.3)
5–11	34.8 (29.3–40.4)	2.9 (2.6–3.2)	23.7 (20.6–26.8)	3.6 (3.3–3.8)	17.3 (15.3–19.3)	4.3 (3.8–4.9)	21.0 (17.9–24.2)	22.2 (20.3–24.2)
12–17	33.3 (28.4–38.2)	3.3 (2.4–4.2)	22.3 (19.4–25.3)	3.4 (3.1–3.6)	16.8 (14.0–19.5)	3.7 (3.3–4.1)	19.9 (15.9–23.9)	24.6 (20.3–28.9)
18–29	17.8 (14.5–21.2)	4.9 (3.1–6.7)	19.7 (13.0–26.3)	7.5 (4.4–10.6)	7.5 (2.3–12.7)	8.2 (5.8–10.6)	15.4 (9.7–21.1)	16.7 (10.7–22.7)
30–64	23.3 (19.6–27.0)	2.5 (2.2–2.9)	12.1 (9.7–14.5)	4.3 (3.1–5.5)	7.1 (5.0–9.2)	7.3 (4.6–10.0)	9.5 (7.6–11.4)	13.4 (9.9–16.8)
≥65	6.8 (5.2–8.4)	2.1 (1.7–2.5)	4.0 (3.3–4.7)	2.3 (2.1–2.6)	2.3 (2.1–2.6)	3.2 (2.9–3.5)	3.7 (3.0–4.4)	4.1 (3.4–4.8)
Sex								
M	21.5 (18.3–24.7)	3.1 (2.7–3.6)	14.5 (12.1–16.8)	4.6 (3.3–5.9)	9.0 (6.6–11.4)	7.2 (4.5–9.8)	10.6 (8.9–12.3)	13.5 (11.1–15.9)
F	19.4 (16.7–22.0)	2.5 (2.1–2.9)	10.9 (9.1–12.8)	3.6 (3.0–4.2)	5.7 (5.0–6.5)	4.8 (3.9–5.6)	10.0 (8.2–11.8)	12.4 (9.4–15.3)
Household size (including participant)								
1	9.9 (6.3–13.6)	1.2 (0.4–1.9)	9.0 (4.1–14.0)	2.8 (1.4–4.2)	3.7 (2.7–4.8)	4.2 (2.8–5.6)	7.8 (4.9–10.8)	10.5 (6.1–14.8)
2	19.3 (14.9–23.7)	2.4 (1.8–2.9)	8.1 (6.7–9.6)	3.1 (2.4–3.7)	5.4 (3.0–7.7)	6.8 (3.5–10.1)	8.0 (5.8–10.2)	10.5 (6.9–14.2)
3	20.7 (17.5–24.0)	3.1 (2.6–3.7)	16.0 (11.9–20.2)	4.5 (2.6–6.4)	7.3 (4.9–9.8)	5.2 (4.0–6.3)	8.9 (7.3–10.5)	14.5 (11.1–17.8)
4	28.6 (24.0–33.2)	3.9 (3.2–4.6)	17.4 (13.7–21.1)	6.3 (4.0–8.6)	12.0 (8.4–15.6)	6.0 (4.9–7.1)	16.0 (12.6–19.4)	16.5 (12.8–20.1)
≥5	23.8 (18.4–29.2)	4.4 (4.0–4.8)	20.8 (15.9–25.7)	5.0 (3.0–7.1)	13.2 (10.2–16.3)	6.9 (5.3–8.5)	17.0 (10.9–23.0)	20.4 (14.0–26.8)
Place of residence								
Attiki	20.4 (18.3–22.4)	2.8 (2.5–3.1)	11.8 (9.5–14.1)	3.3 (2.8–3.9)	7.3 (4.9–9.6)	5.3 (4.3–6.4)	10.1 (8.1–12.0)	13.7 (11.0–16.4)
Thessaloniki	-	-	13.7 (9.7–17.8)	3.3 (2.6–3.9)	6.8 (5.1–8.5)	4.9 (3.6–6.3)	10.3 (7.6–13.1)	14.2 (6.0–22.5)
Other regions	-	-	13.0 (10.8–15.2)	4.7 (3.5–5.9)	7.4 (5.8–9.0)	6.4 (4.2–8.6)	10.4 (8.6–12.2)	12.3 (9.6–15.0)

*Shaded columns indicate lockdown periods.

Sensitivity Analysis Censoring at 100 Contacts

Appendix Table 3. Mean (95% CI) daily number of contacts per participant without censoring and with censoring at 100 contacts, Greece, January 2020-October 2021*

Study Period	January 2020	March–April 2020	September 2020	November – December 2020	February 2021	April 2021	May–June 2021	September–October 2021
No. participants	602	602	1,203	1,203	1,200	1,201	1,202	1,200
No. contacts without censoring	20.4 (18.3–22.4)	2.8 (2.5–3.1)	12.7 (11.2–14.1)	4.1 (3.4–4.8)	7.3 (6.1–8.5)	5.9 (4.6–7.3)	10.3 (9.0–11.5)	12.9 (11.0–14.8)
No. contacts with censoring at	19.7 (17.9–21.5)	2.8 (2.5–3.1)	11.8 (10.7–13.0)	3.9 (3.4–4.5)	6.7 (6.0–7.3)	5.4 (4.8–6.0)	9.7 (8.7–10.7)	11.5 (10.3–12.7)

Study Period	January 2020	March–April 2020	September 2020	November – December 2020	February 2021	April 2021	May–June 2021	September–October 2021
100 contacts								

*The estimates are adjusted for the age and sex distribution of the population of Greece based on the first-level NUTS regions. Shaded columns indicate lockdown periods.

Number of Contacts by Survey Period and Location

Appendix Table 4. Mean (95% CI] daily number of contacts per participant by survey period and location, Greece, January 2020-October 2021*

Study Period	January 2020	March–April 2020	September 2020	November–December 2020	February 2021	April 2021	May–June 2021	September–October 2021
Home	2.4 (2.2–2.7)	2.0 (1.8–2.1)	2.2 (2.0–2.3)	2.0 (1.9–2.1)	2.0 (1.9–2.1)	2.2 (2.1–2.4)	2.1 (2.0–2.2)	2.1 (2.0–2.3)
Work	8.5 (6.6–10.4)	0.5 (0.3–0.7)	5.9 (4.5–7.4)	1.1 (0.6–1.7)	2.2 (1.1–3.2)	2.5 (1.2–3.9)	4.1 (3.0–5.2)	5.6 (3.8–7.3)
School	5.1 (4.1–6.0)	-	3.1 (2.6–3.6)	-	2.1 (1.7–2.5)	-	2.7 (2.1–3.3)	3.4 (2.7–4.0)
Other (Leisure, transport, etc.)	5.2 (4.3–6.1)	0.4 (0.3–0.5)	2.0 (1.7–2.2)	1.1 (0.7–1.6)	1.3 (0.8–1.8)	1.4 (1.2–1.5)	1.8 (1.5–2.1)	2.3 (1.7–2.9)
Adults								
Home	2.2 (1.9–2.5)	1.8 (1.7–1.9)	2.0 (1.9–2.2)	1.8 (1.7–1.9)	1.7 (1.6–1.8)	2.0 (1.9–2.1)	1.9 (1.8–2.0)	1.9 (1.7–2.0)
Work	10.3 (8.0–12.5)	0.6 (0.3–0.9)	7.2 (5.5–8.9)	1.4 (0.7–2.0)	2.6 (1.3–3.9)	3.1 (1.5–4.7)	4.9 (3.6–6.2)	6.7 (4.6–8.8)
Other (Leisure, transport, etc.)	5.4 (4.4–6.4)	0.5 (0.4–0.6)	1.9 (1.6–2.2)	1.2 (0.7–1.8)	1.4 (0.8–2.0)	1.4 (1.2–1.7)	1.8 (1.5–2.2)	2.4 (1.7–3.1)
Children								
Home	3.6 (3.4–3.8)	2.9 (2.5–3.2)	2.7 (2.5–2.9)	3.1 (2.9–3.3)	3.1 (3.0–3.3)	3.3 (3.2–3.5)	3.4 (3.2–3.6)	3.4 (3.1–3.7)
School	24.2 (20.8–27.7)	-	16.2 (14.4–18.1)	-	10.5 (9.2–11.8)	-	13.1 (11.0–15.3)	16.3 (14.2–18.4)
Other (Leisure, transport, etc.)	4.2 (2.5–6.0)	0.1 (0.0–0.2)	2.3 (1.8–2.9)	0.4 (0.3–0.6)	1.1 (0.5–1.6)	1.0 (0.7–1.3)	1.6 (1.0–2.1)	1.6 (1.1–2.1)

*The sum of contacts at home, work, school and other settings does not necessarily add to the total number of contacts, as some participants may have had contacts with the same person in more than one settings on a specific day. Shaded columns indicate lockdown periods.

Vaccination Coverage by Survey Period in Greece

Appendix Table 5. Cumulative uptake of at least one vaccine dose by age groups in Greece*

Survey period	Week number	<18	18–24	25–49	50–59	≥60
Mid-January 2020		0.0%	0.0%	0.0%	0.0%	0.0%
March 31–April 7, 2020		0.0%	0.0%	0.0%	0.0%	0.0%
Late September 2020		0.0%	0.0%	0.0%	0.0%	0.0%
November 17–December 3, 2020		0.0%	0.0%	0.0%	0.0%	0.0%
February 1–18, 2021	2021-W06	0.0%	**0.3%**	**2.3%**	**2.9%**	**8.1%**
April 1–12, 2021	2021-W14	0.0%	**0.9%**	**4.4%**	**5.8%**	**38.9%**
May 17–June 5, 2021	2021-W21	0.0%	**2.8%**	**22.2%**	**48.3%**	**67.9%**
September 28–October 15, 2021	2021-W40	**9.9%**	**57.9%**	**66.9%**	**75.3%**	**79.6%**

*Data from the European Centre for Disease Prevention and Control COVID-19 Vaccine Tracker (https://vaccinetracker.ecdc.europa.eu/public/extensions/COVID-19/vaccine-tracker.html#age-group-tab).

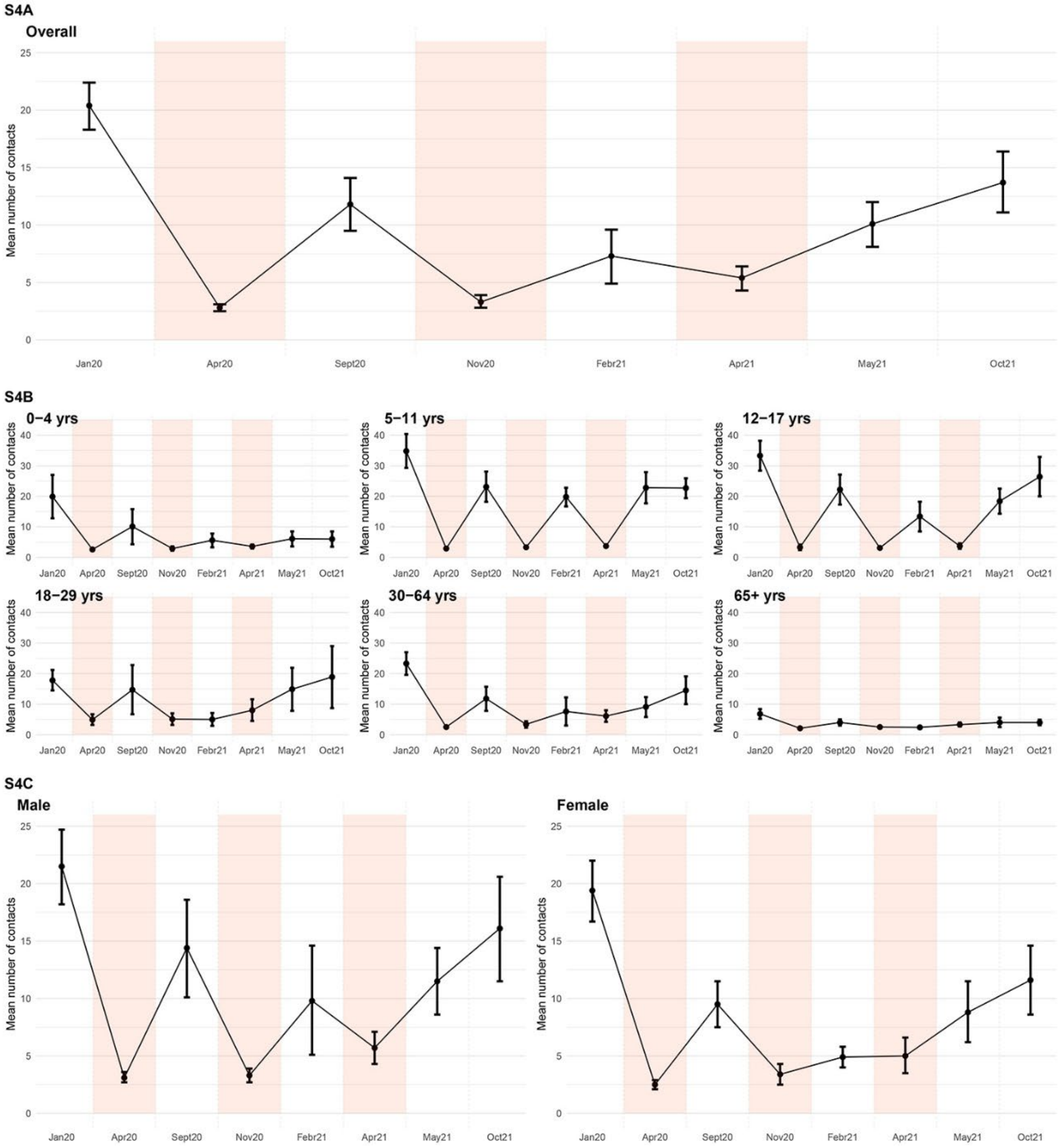

Appendix Figure 1. Sensitivity analysis including only participants living in Attica: Mean daily number of recorded social contacts per participant living in Attica in study of social contact patterns and age-mixing before and during COVID-19 pandemic, Greece, January 2020–October 2021. Data are shown for 8 social contact data collection periods overall (A), by age group (B), and by sex (C). Error bars mark 95% CIs. Shaded areas indicate lockdown periods.

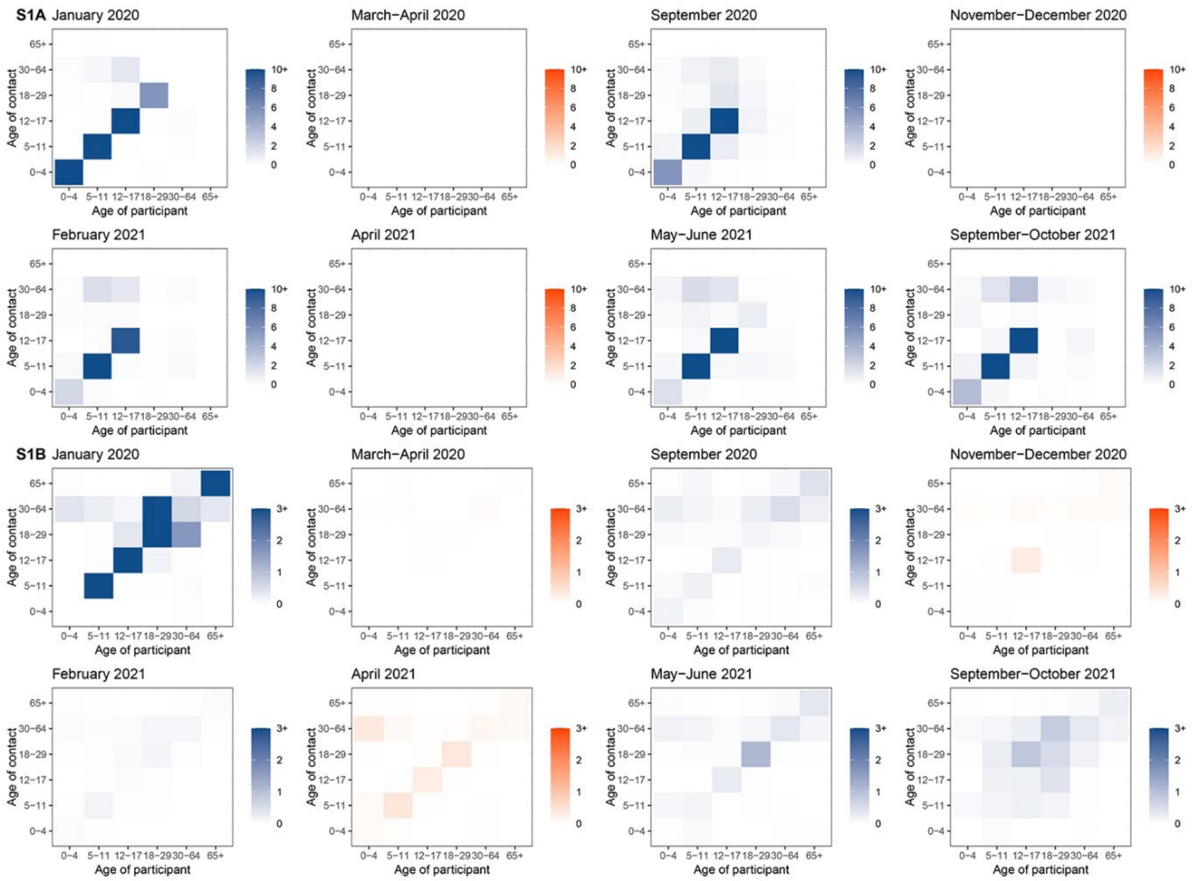

Appendix Figure 2. Age-specific contact matrices A) at school, B) during leisure activities in study of social contact patterns and age-mixing before and during COVID-19 pandemic, Greece, January 2020-October 2021. Each cell represents the average daily number of reported contacts, stratified by the age group of the participants and their corresponding contacts. Gradient palettes were used to color contact matrices (orange-red indicates lockdown periods, and blue indicates prepandemic period and periods with relaxed measures).

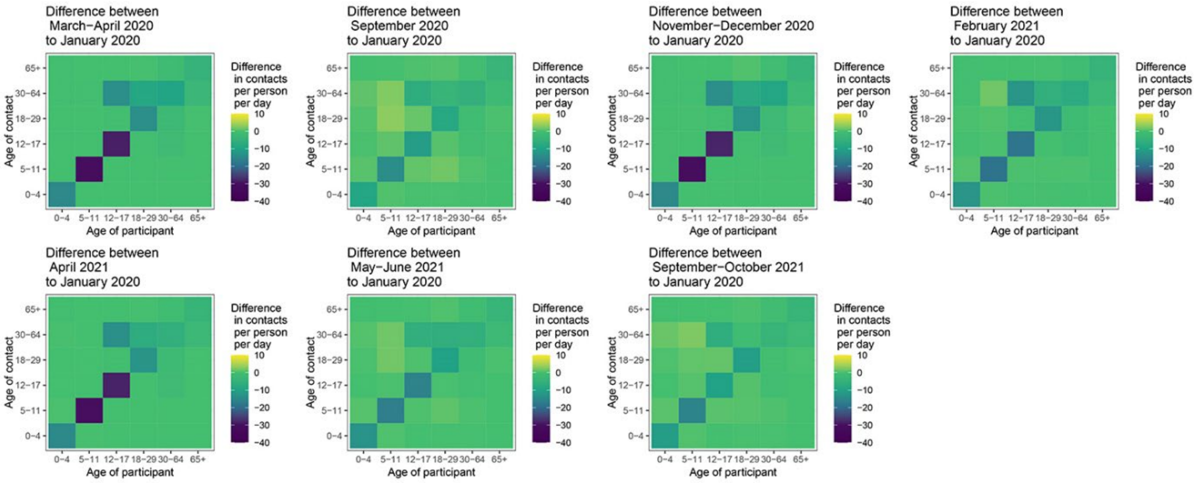

Appendix Figure 3. Absolute difference in the daily number of contacts between each of the seven study periods during the pandemic (March 2020-October 2021) and the prepandemic period (January 2020) in Greece.

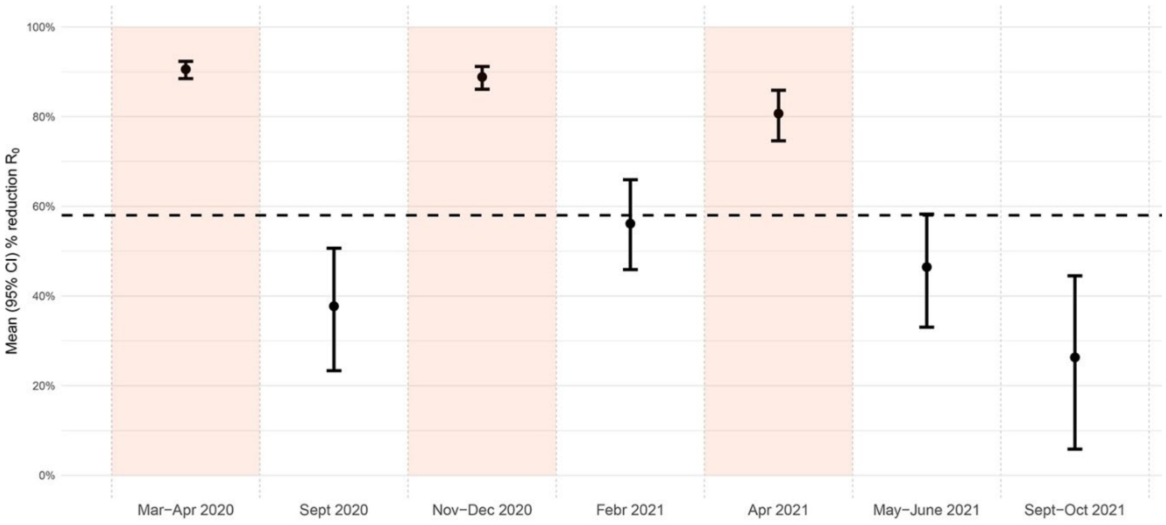

Appendix Figure 4. Sensitivity analysis including only participants living in Attica: Mean reduction in R_0 caused by physical distancing measures during COVID-19 pandemic (March 2020-October 2021) compared with prepandemic period (January 2020) in Attica, Greece. R_0 reduction was obtained by comparing social contacts data from each study period during the pandemic to the prepandemic period (January 2020). Error bars mark 95% CIs. Shaded areas indicate lockdown periods. Dashed horizontal line indicates the minimum reduction needed to bring R_0 to <1, assuming R_0 is equal to 2.38 (based on Ref. 15 of the paper). R_0, basic reproduction number.

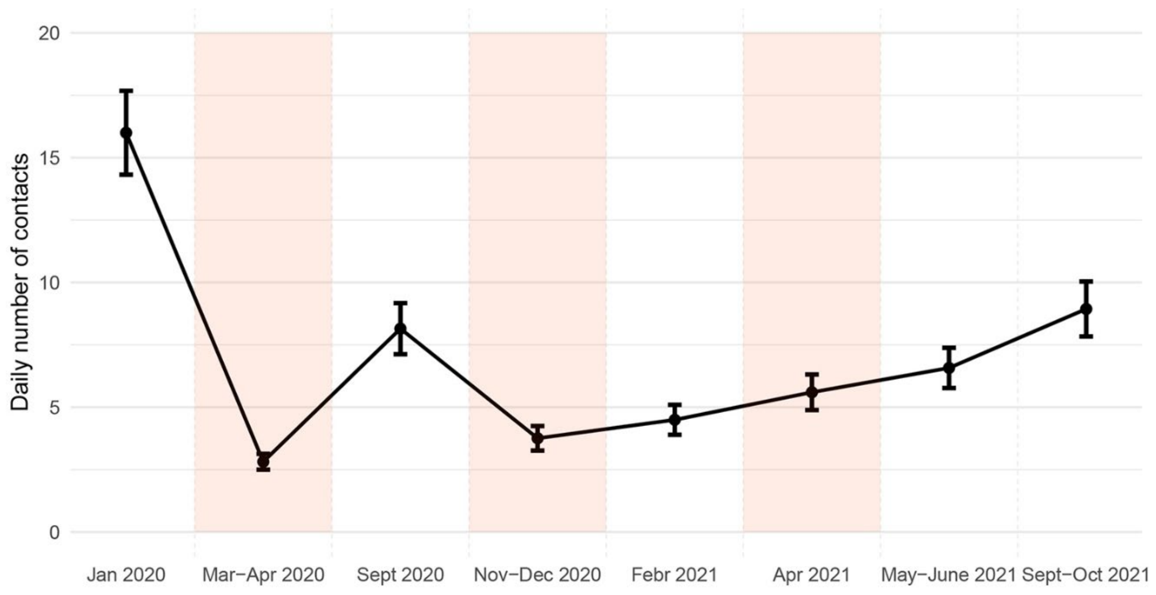

Appendix Figure 5. Sensitivity analysis including only participants living in Attica: Adjusted average predictions of the number of contacts of adult participants living in Attica in study of social contact patterns and age-mixing before and during COVID-19 pandemic, Greece, January 2020-October 2021 (N = 3,779). Data are shown for study period. Results from negative binomial generalized linear mixed models with random intercepts at the individual level fitted on social contact data collected across 8 periods in Greece through cross-sectional surveys. Error bars mark 95% CIs. Shaded areas indicate lockdown periods.

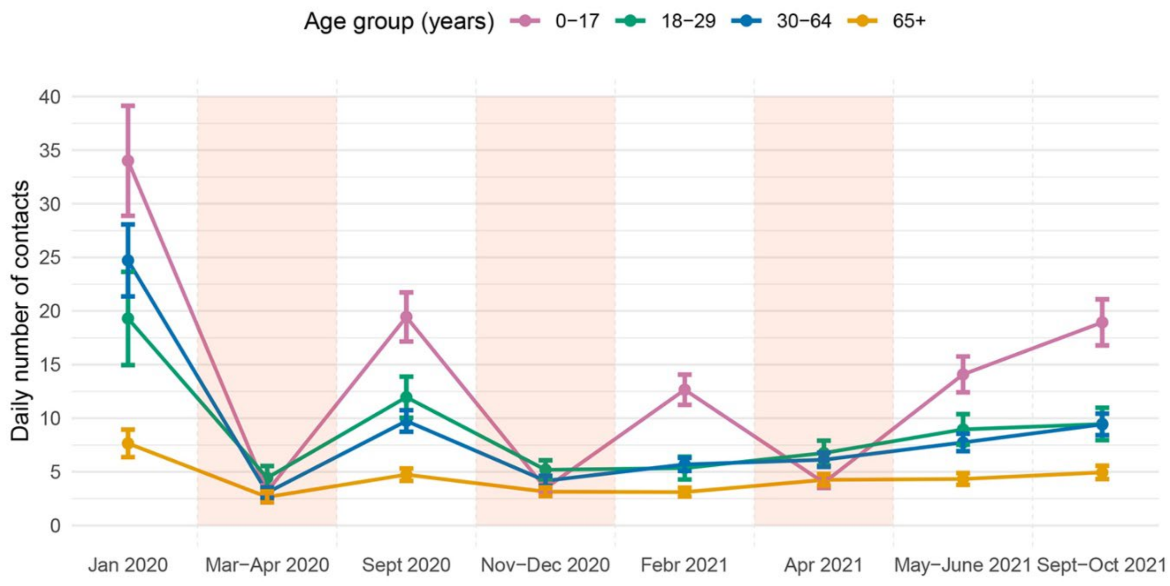

Appendix Figure 6. Adjusted average predictions of the number of contacts of participants of all ages (sensitivity analysis including children and adolescents and with a more detailed age breakdown of adults) in study of social contact patterns and age-mixing before and during COVID-19 pandemic,

Greece, January 2020-October 2021 (N = 8,413). Data are shown for study period according to the age group of participants. Results from negative binomial generalized linear mixed models with random intercepts at the individual level fitted on social contact data collected for 8 periods in Greece through cross-sectional surveys. Error bars mark 95% CIs. Shaded areas indicate lockdown periods.
